# Effects of soil properties on the uptake of pharmaceuticals into earthworms^☆^

**DOI:** 10.1016/j.envpol.2016.03.044

**Published:** 2016-06

**Authors:** Laura J. Carter, Jim J. Ryan, Alistair B.A. Boxall

**Affiliations:** a Environment Department, University of York, Heslington, York, YO10 5DD, UK; b EHS Technical CoE, GlaxoSmithKline, Ware, SG12 0DP, UK

**Keywords:** *Eisenia fetida*, Minimised design, Soil properties, Bioaccumulation

## Abstract

Pharmaceuticals can enter the soil environment when animal slurries and sewage sludge are applied to land as a fertiliser or during irrigation with contaminated water. These pharmaceuticals may then be taken up by soil organisms possibly resulting in toxic effects and/or exposure of organisms higher up the food chain. This study investigated the influence of soil properties on the uptake and depuration of pharmaceuticals (carbamazepine, diclofenac, fluoxetine and orlistat) in the earthworm *Eisenia fetida*. The uptake and accumulation of pharmaceuticals into *E. fetida* changed depending on soil type. Orlistat exhibited the highest pore water based bioconcentration factors (BCFs) and displayed the largest differences between soil types with BCFs ranging between 30.5 and 115.9. For carbamazepine, diclofenac and fluoxetine BCFs ranged between 1.1 and 1.6, 7.0 and 69.6 and 14.1 and 20.4 respectively. Additional analysis demonstrated that in certain treatments the presence of these chemicals in the soil matrices changed the soil pH over time, with a statistically significant pH difference to control samples. The internal pH of *E. fetida* also changed as a result of incubation in pharmaceutically spiked soil, in comparison to the control earthworms. These results demonstrate that a combination of soil properties and pharmaceutical physico-chemical properties are important in terms of predicting pharmaceutical uptake in terrestrial systems and that pharmaceuticals can modify soil and internal earthworm chemistry which may hold wider implications for risk assessment.

## Highlights

• Uptake of pharmaceuticals into earthworms is influenced by soil parameters.
• Presence of pharmaceuticals in the terrestrial environment influences soil pH.
• Uptake of pharmaceuticals by earthworms changes internal earthworm pH.

## Teaser

The uptake of pharmaceuticals into soil invertebrates is dependent on the complex interplay between pharmaceutical physico-chemical properties and soil parameters.

## 1 Introduction

Following use, pharmaceuticals are typically excreted to the sewage system and are then transported to wastewater treatment plants. As many pharmaceuticals are resistant to degradation in wastewater treatment processes they will be present in the wastewater treatment effluents and in the sludge by-products (Jelic et al., 2011). The land application of sewage sludge (biosolids) as a fertiliser and use of reclaimed waste water for irrigation purposes therefore provides a route of entry for pharmaceuticals into the terrestrial environment (Dalkmann et al., 2012, Duran-Alvarez et al., 2009, Kinney et al., 2006a, Kinney et al., 2006b, Siemens et al., 2008). Concerns have therefore been raised over the potential uptake of pharmaceuticals into terrestrial organisms and the potential effects on soil-dwelling organisms and organisms that feed on these (Arnold et al., 2014). A handful of studies have recently demonstrated that pharmaceuticals can be taken up from soils and accumulate in invertebrates such as earthworms (Berge and Vulliet, 2015, Carter et al., 2014b, Kinney et al., 2008).

Earthworms are key terrestrial invertebrates with respect to the role they have in maintaining a fertile soil environment (Edwards, 2004). Earthworms are also a key food source for many predator species such as birds. Understanding the uptake of chemicals into earthworms is therefore not only a prerequisite to understanding the risks chemicals pose to earthworm populations, but also the potential effects of secondary poisoning on predators. Earthworms are at the base of many food chains and thus if chemicals are taken up into the earthworms they can facilitate the movement of chemicals into the food web via bioaccumulation and bio-magnification processes (Shore et al., 2014).

We have previously investigated the uptake and depuration kinetics of four pharmaceuticals in the earthworm, *Eisenia fetida* (Carter et al., 2014b). Pore-water based bioconcentration factors (BCFs) increased in the order of carbamazepine < diclofenac < fluoxetine < orlistat and ranged between 2.2 and 51.5. This study highlighted that, unlike neutral organic compounds, uptake of ionisable pharmaceuticals was not driven by the hydrophobicity (log K_ow_) of the chemical alone. Our previous study exposed the earthworms to pharmaceutical residues in one soil type only. It is well known that pharmaceuticals can behave very differently in different soil types (Drillia et al., 2005, Monteiro and Boxall, 2009). For example, distribution coefficients (K_d_) between soil particles and soil pore waters are known to vary by several orders of magnitude for a range of pharmaceuticals in soils with varying properties (Kodesova et al., 2015, ter Laak et al., 2006). As the distribution of pharmaceuticals between the soil and pore water influences the bioavailable fraction of these chemicals and thus uptake by earthworms, it is therefore likely that uptake of pharmaceuticals could also vary significantly across soils.

However, knowledge of the relationships between soil properties and pharmaceutical uptake in terrestrial species is very limited. There is therefore a real need to generate data on the uptake of pharmaceuticals into terrestrial invertebrates from soils with different characteristics in order to identify key drivers affecting uptake. This will help to develop uptake modelling approaches for use in environmental risk assessment. Therefore, in this study we build upon our previously published results demonstrating pharmaceutical uptake by the earthworm *Eisenia fetida* in a single soil type (Carter et al., 2014b) and explore the effects of soil properties on the uptake and depuration of pharmaceuticals in order to help elucidate the relationships between soil properties and uptake. The study focused on one acidic (diclofenac), one basic (fluoxetine) and two neutral (carbamazepine and orlistat) pharmaceuticals, from a variety of therapeutic uses and covering a range of physico-chemical properties (e.g. log K_ow_ 2.25–8.19) (Table 1). With the exception of orlistat, these pharmaceuticals have been previously detected in wastewater irrigated soils in concentrations <7 μg/kg and therefore it is important to understand the potential uptake of these chemicals by soil dwelling organisms. To help explain any potential differences in uptake and depuration, parallel studies were performed to assess the fate and distribution of the study pharmaceuticals in test soils.

## 2 Materials and methods

### 2.1 Pharmaceuticals and reagents

All studies were performed using ^14^C labelled compounds. Radiolabeled fluoxetine [methyl-^14^C] and carbamazepine [carbonyl-^14^C] were obtained from American Radiolabeled Chemicals (St. Louis, MO, USA), diclofenac [U – ^14^C] was obtained from Perkin Elmer (Boston, MA, USA) and orlistat [tridecanyl-2-^14^C] was provided by GlaxoSmithKline (GSK) (Middlesex, UK). Physico-chemical properties and specific activities for the pharmaceuticals can be found in Table 1. Acetonitrile (99.9%), methanol (99.9%) and ethyl acetate (99.9%) were obtained from Fisher Scientific (Loughborough, UK).

### 2.2 Test soils

Five standard test soils were obtained from LUFA Speyer (Speyer, Germany). The soils, 2.1, 2.3, 2.4, 5M and 6S, included clay loam, silty sand and loamy sand varieties and were chosen to provide a range of soil characteristics including varying soil pH, organic carbon content, cation exchange capacity and particle size distributions (Table 2). Soils were air dried and sieved to 2 mm prior to testing to ensure homogeneity.

### 2.3 Test organism

*E. fetida* were obtained from Blades Biological Ltd. (Kent, UK) and cultured in a medium of peat and cow manure (50:50) (Dean's Garden Centre, York, UK), kept moist with deionised water at room temperature (20 ± 3 °C). The earthworms were fed twice weekly with homogenised mashed potato powder. *E. fetida* were obtained from a single species culture and cultures were maintained for at least four generations prior to use in the uptake studies. The lipid content of *E. fetida*, determined using the method of Folch et al., (Folch et al., 1957), was 5.11 ± 0.29% (wet weight) (Carter et al., 2014b).

### 2.4 Fate studies

For each pharmaceutical, triplicate beakers of each soil (2.1, 2.3, 2.4, 5M and 6S) (35 ± 1 g) were prepared to sample at eight time points (0 and 6 h, 1, 3, 7, 10, 14 and 21 d) where pore water and soil samples would be analysed for radioactivity and pH. Detailed sample preparation and analysis techniques can be found in Carter et al. (2014b). Briefly, labelled pharmaceuticals were added, individually, to each of the five soils using 125–165 μl of a carrier solvent to create nominal concentrations of 26, 25, 28 and 44 μg/kg of carbamazepine, diclofenac, fluoxetine and orlistat respectively. For carbamazepine and fluoxetine, ethanol was used as the carrier solvent; for diclofenac, methanol was used and orlistat was applied in acetonitrile. After spiking, each test beaker was left for 2 h and then mixed by hand to create an even distribution of the pharmaceutical within the sample. Following spiking and mixing, the carrier solvents were allowed to evaporate from the test beakers for 48 h. Blank and solvent controls were also prepared. The moisture content of all soils was adjusted, and maintained at 40–60% of the maximum water holding capacity (MWHC) by addition of deionised water on a daily basis. All experiments were undertaken at 20 ± 2 ^°^C, using a 16:8 light/dark cycle [600 lx] and 60% humidity.

### 2.5 Uptake and depuration studies

The uptake and depuration studies followed the ‘minimised’ approach described in Carter et al. (2014a). The experiments consisted of exposing a single earthworm to each pharmaceutical in each of the five soil types. There were six replicates per treatment. Soils were prepared in glass jars (50 ± 1 g) and spiked with the four pharmaceuticals at similar concentrations and following similar methods to those in the fate studies. In total there were 60 spiked soils per pharmaceutical compound (12 spiked soils × 5 soil types). For each soil type, blank (earthworm exposed in uncontaminated soil; n = 6) and solvent controls (earthworm exposed in soil spiked with solvent only; n = 6) were prepared. Adult *E. fetida* (ranging in mass from 220 to 450 mg wet weight, and with average mass 299 mg (standard deviation 54 mg)) (Supplementary Table 1) were then added to each test beaker after having been acclimatised under experimental conditions for 48 h in non-treated test soil. After addition to the soil surface, the time it took for each earthworm to completely burrow into the soil was also noted. Earthworm beakers were incubated under controlled conditions and moisture adjustments were performed as reported in the fate study. For each pharmaceutical treatment in each soil type, six replicates were sampled at the end of the uptake period (21 d) and six at the end of the depuration phase (42 d). *E. fetida* were then removed from the vessels, and transferred to moist filter paper for 24 h to allow them to purge their guts. The earthworms were then frozen at −20 °C until analysis.

### 2.6 Preparation of samples for analysis

Pore-water was extracted from the soils using a centrifugation method and soil and earthworms were extracted using liquid extraction methods similar to those outlined in Carter et al. (2014b) and are provided in the Supplementary materials together with the extraction recoveries (Supplementary Table 2). Before *E. fetida* extraction, the internal pH of each worm was measured using an Orion™ pH microelectrode (Thermo Scientific, UK). Each worm was dissected across the segments from the anterior to the posterior. The pH probe was then placed into the earthworm tissue avoiding internal organs, to determine internal tissue pH. Soil pH measurements were also obtained by preparing a soil solution from each test vessel. Methanol, ethyl acetate, acetonitrile:water (70:30 v/v) and acetonitrile were then used as solvents in the *E. fetida* and soil extractions for carbamazepine, diclofenac, fluoxetine and orlistat respectively. Average recoveries ranged from 72.4 to 94.7% for the pharmaceuticals in the five different soil types (detailed recovery information provided in the Supporting Information). Recoveries ranged from 86.3 (fluoxetine) to 100.9% (carbamazepine and diclofenac) for the earthworm extraction methods. As previous work has demonstrated that orlistat and diclofenac form irreversibly bound residues with soil (Carter et al., 2014b); combustion analysis of these samples was also performed using a Perkin Elmer 307 Sample Oxidiser (see the Supporting Information for a description of the soil combustion procedure).

#### 2.6.1 Liquid scintillation counting

Radioactivity in soil pore water, soil and earthworm extracts was determined using Liquid Scintillation Counting (LSC) using a Beckman LS 6500 LSC counter (Beckman Coulter Inc., Fullerton, CA, USA). Samples were counted three times for 5 min. Counts were corrected for background activity by using blank controls. Counting efficiency and colour quenching were corrected for using the external standard ratio method.

### 2.7 Calculating BCF–kinetic modelling

Previous work was able to elucidate that approximately 23% of the soil gut contents remained in the *E. fetida* gut after 24 h of purging. In the present study, after measuring the amount of gut contents eliminated during the 24 h period, the amount of soil remaining in the gut was calculated and then a correction factor was applied to the measured radioactivity in the earthworm extracts to account for soil-associated pharmaceuticals present in the gut and ensure that the analysis focussed on the tissue concentrations only (see Carter et al. (2014b) for more detail). Measured radioactivity in the *E. fetida* extracts were then used to calculate uptake and depuration rates for each study compound in each soil type using Equation (1) and Equation (2). For a full explanation of BCF calculations see Carter et al. (2014a).

(1) $$k_{2}=(\ln\;Ct_{1}-\ln\;Ct_{2})/td$$

(2) $$k_{1}=k_{2}*C_{t_{1}}/C_{pw}\left(1-e^{-k_{2}t_{u}}\right)$$

Where *k*_*2*_ is the depuration rate constant, *k*_*1*_ is the uptake rate constant, *C*_*pw*_ is average pore water concentration during exposure phase (n = 3), *C*_*t1*_ and *C*_*t2*_ are the average *E. fetida* concentrations after the uptake and depuration phases respectively (n = 6) and *t*_*u*_, *t*_*d*_ are the length of uptake and depuration period respectively. The uptake and depuration rates were then used to estimate pore water based kinetic bioconcentration factors (Equation (3)).

(3) $${\text{BCF}}_{\text{minimised}}=k_{1}/k_{2}$$

Soil based bioaccumulation factors (BSAF) were estimated from the pore water based BCFs for all pharmaceuticals using soil water partition coefficients (K_d_) calculated from fate studies (Equation (4)). A soil water partition coefficient, defined as a ratio of the concentration of each pharmaceutical in the soil and pore water during the 21 d fate study, was determined for each replicate, at each sampling point for all soils. An average K_d_ across all replicates and sampling points was then calculated for each pharmaceutical in each soil type (Table 2).

(4) $${\text{BSAF}}_{\text{soil}}={\text{BCF}}_{\text{minimised}}/{\text{K}}_{\text{d}}$$

Regression analysis was then performed to compare BSAF values and soil properties and BCF values to pore water properties.

### 2.8 Statistical analysis

Statistical analysis was performed using SigmaPlot (v.12) with a significance level of 0.05. Prior to all tests, the data were tested for normality and equal variance using a Shapiro–Wilk and Levene–Mediane test; respectively to ensure the ANOVA conditions were satisfied. If the normality test failed then the one-way ANOVA was instead performed on ranks. Firstly, data on burrowing times were tested against the control treatment using a one-way ANOVA, to assess any pharmaceutical effects on earthworm behaviour. A two-way analysis of variance (ANOVA) was performed, keeping study type (blank or treatment) as repeated and time as a variable factor. Endpoints tested included the differences in soil pH across time and in comparison to control samples, additional pair-wise comparisons of the data were performed according to the Holm-Sidak method. Further two-way ANOVAs were performed to check differences in internal *E. fetida* pH exposed in the same soil but under different pharmaceutical treatments (including controls) at both the end of the uptake and depuration phases.

## 3 Results

### 3.1 Pharmaceutical fate in soils

**Fig. 1:**
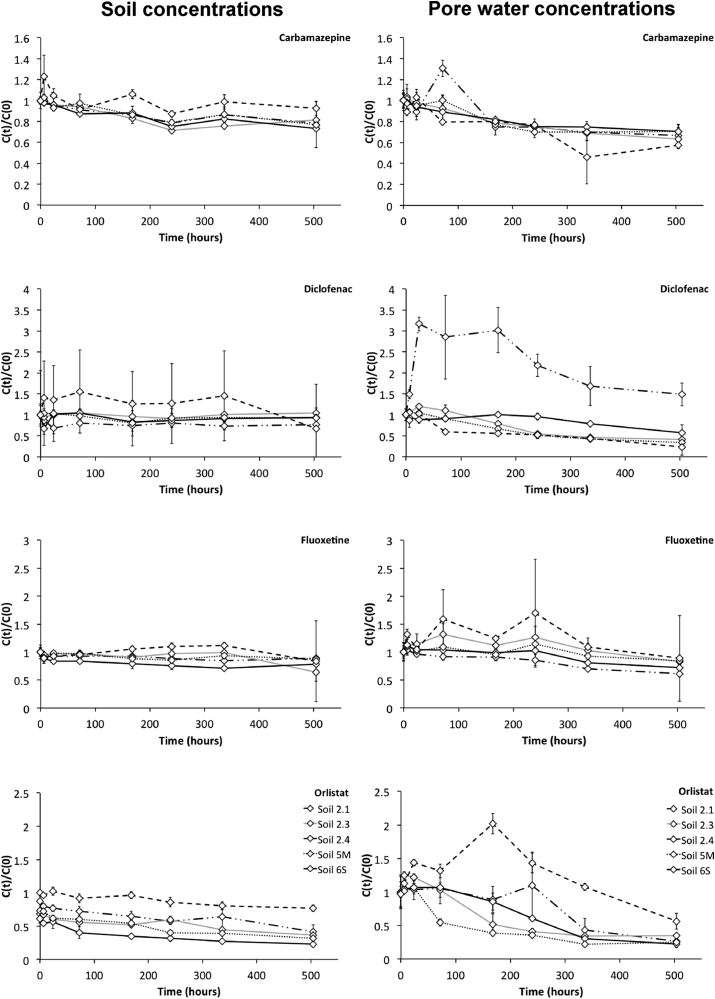
Dissipation of radioactivity measured in carbamazepine, diclofenac, fluoxetine and orlistat studies in soil and pore water throughout 21 day in five different soil types 2.1 (dash and dotted); 2.3 (grey); 2.4 (black); 5M (dotted) and 6S (dash). Average C(t)/C(0) ratio provided with ±standard deviation, where C(t) is concentration at time of sampling throughout the fate study and C(0) is concentration at 0 d.

**Fig. 2:**
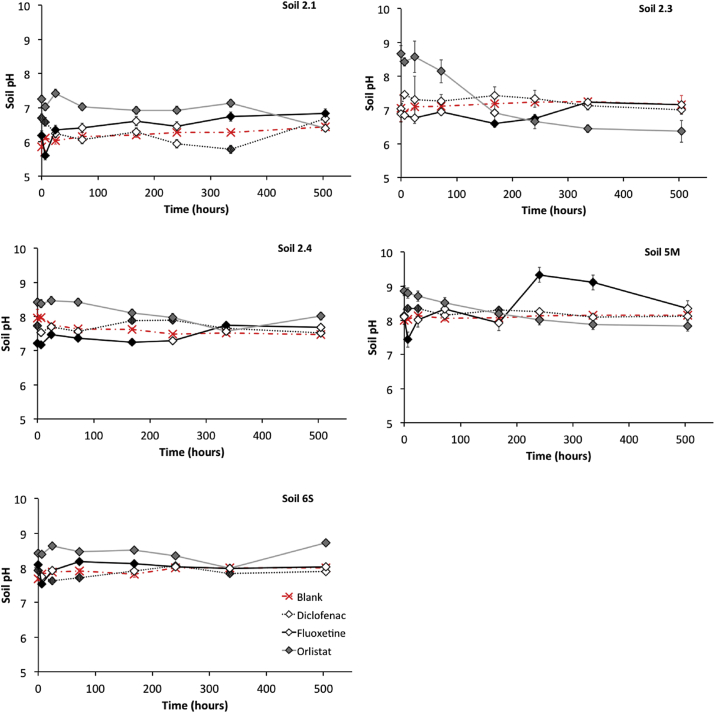
Changes in soil pH over time for different soil types under diclofenac, fluoxetine and orlistat exposure. Average measurements provided (n = 3) together with standard deviation. Results which are significantly different to the control measurement are denoted by a filled diamond and results not significantly different to the control are an indicated by an open diamond (*p* < 0.05).

Measurements of extractable radioactivity in the soil and pore water changed over time and these changes appear to be dependent on pharmaceutical compound and in a number of cases, on soil type (Fig. 1). It is important to note that as the fate experiments were performed without earthworms, their presence might have affected the fate differently. Nevertheless the results can contribute to our understanding on the behaviour of pharmaceuticals in the soil and pore water over time and provide a likely indication as what the earthworms would be exposed to. In most soil types, measured radioactivity in soil tended to decrease after 1d. Carbamazepine was fairly persistent in all soil types whilst initial results showed rapid dissipation of diclofenac and orlistat from the test beakers. However, combustion analysis confirmed the formation of non-extractable (bound) residues (NER's) in both the diclofenac and orlistat studies. NER fractions increased over time reaching a maximum 49.9% of the total radioactivity in soil 2.4 and 97.4% in soil 2.3 of for the orlistat and diclofenac exposures respectively (see Supplementary Figure 1 and 2 for additional information on the mass balance). Changes in soil pH over time, and in comparison to the controls were noted over 21 d as a result of the presence of pharmaceuticals in the soil (diclofenac, fluoxetine and orlistat) in the soil matrix (*p* < 0.05) (Fig. 2). In comparison to the control soils, soil pH was overall significantly different in all soil five types for the fluoxetine and orlistat exposures and in the diclofenac treatment with the exception of soil 2.3 (silty sand) (*p* < 0.05). Whilst these changes appear to be influenced by soil type it is important to note that changes in soil pH were not consistent over time.

Concentrations of the pharmaceuticals in the pore-water differed to a greater extent, depending on soil type, in comparison to the soil concentrations (Fig. 1). Soil 2.1 (silty sand) generally had the highest pore water concentrations for all pharmaceuticals while the clayey loam soil (soil 2.4) generally had the lowest concentrations. From 10 d onwards, pore-water concentrations tended to decrease in all soil types especially for diclofenac, fluoxetine and orlistat. This was most evident in the silty sand soil (soil 2.1) for all pharmaceuticals.

The soil–water distribution (K_d_) for the individual pharmaceuticals varied across soil types with K_d_ values for carbamazepine ranging from 1.34 to 4.45 L/kg, diclofenac ranging from 5.63 to 18.37 L/kg, fluoxetine ranging from 55.48 to 71.44 L/kg and orlistat ranging from 28.99 to 110.01 L/kg (Table 3). Over the initial 10 d of the uptake phase, orlistat became less strongly bound to the soil as the amount recovered in the solvent extraction increased whilst the combustion analysis concentrations decreased.

### 3.2 Earthworm uptake

Overall mortality during the experiment was less than 4% across all exposure scenarios and whilst the mean earthworm mass did increase over the course of the experiment this was less than 20%. No significant difference in the burrowing times between treatments and controls was noted for any of the soil types (*p* > 0.05), and therefore based on these findings effects of toxicity on uptake can likely be excluded. All four study compounds were found to be taken up from all soil types into *E. fetida* (Table 3). Based on previous research using *E. fetida*, the uptake measured in the carbamazepine and fluoxetine studies is likely to be that of the parent compound (Carter et al., 2014b). The radioactivity measured in the diclofenac study is however more likely to be a transformation product as the parent compound was unable to be detected in *E. fetida* samples exposed to unlabelled diclofenac (Carter et al., 2014b). The transformation of pharmaceuticals can however be influenced by environmental factors, such as pH and temperature (Fatta-Kassinos et al., 2011). Therefore, it is important to note that in this study earthworm exposure in different soil types may have resulted in variable chemical metabolism in the soil or *E. fetida*. The fluoxetine treatment had the greatest uptake rate (*k*_*1*_) in all soils (0.96–2.35 mL/g d^−1^), whilst the carbamazepine treatment had the fastest depuration rate (*k*_*2*_) in all five soils (0.16–0.24 d^−1^) (Table 3). This is comparable to previous work with *E. fetida* in a single soil type (Carter et al., 2014b). Highest pore water-based BCFs were observed for orlistat (<115.88) and the smallest BCFs were seen for carbamazepine. BCFs of the individual compounds were found to differ across soil types, with greatest variability observed for diclofenac (7.02–69.57) and orlistat (30.50–115.88), whereas smaller variability of the BCFs was noted for fluoxetine (14.09–20.42) and carbamazepine (1.05–1.61) (Table 3).

**Fig. 3:**
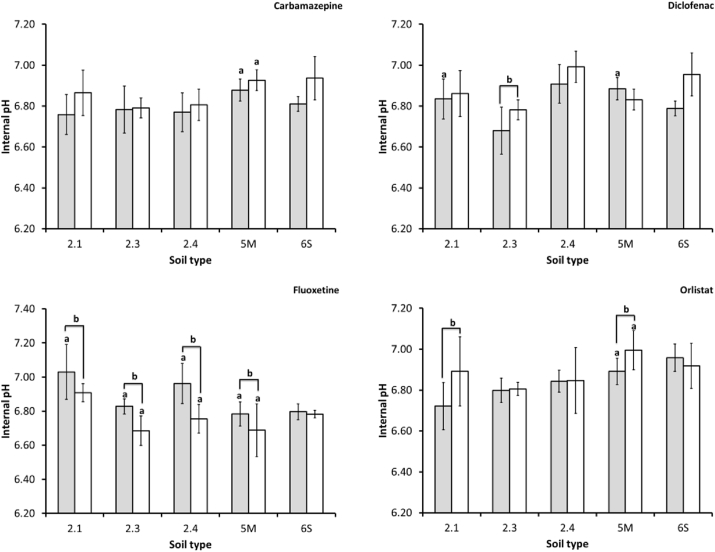
Average measured internal pH in *E. fetida* in different soil types at the end of the uptake (grey) and depuration (white) phase after exposure to carbamazepine, diclofenac fluoxetine and orlistat (n = 6, ±standard deviation). Measurements which are significantly different to the control are denoted by an ‘a’ and where there is a statistically significant difference between internal pH in uptake phase and depuration phase these are denoted by a ‘b’ (*p* < 0.05).

BSAFs were generally low (<2), especially for carbamazepine and fluoxetine. Similarly to the pore-water based BCFs, the diclofenac exposure resulted in the largest range of BSAFs, up to 12.36 in the loamy sand soil (soil 5M). There was a significant difference in internal *E. fetida* pH after exposure to pharmaceuticals in comparison to control earthworms (*p* < 0.05). However, this was not true for all soil types as there was no significant interaction between treatment or time with internal earthworm pH exposed in soil 6S (clayey loam) (Fig. 3). Fluoxetine appears to have the strongest influence on internal earthworm pH as this exposure resulted in significant differences to the control for all soils, with the exception of the clayey loam soil (soil 6S) (*p* < 0.05). Significant differences were also observed between measurements made in earthworms sampled at the end of the uptake and depuration phase respectively (Fig. 3). For the fluoxetine exposure, transferring the earthworms to clean soil appears to reduce the internal pH (*p* < 0.001) whereas for the remaining treatments earthworm pH increased in those earthworms sampled at the end of the depuration phase (*p* < 0.05). Interestingly, not only does the internal pH change between different soil types it was also significantly different between different pharmaceutical treatments in a single soil type at the end of the uptake phase (except soil 5M (loamy sand) and 6S (clayey loam)) (*p* < 0.05) and the end of the depuration phase (except soil 2.1 (silty sand) and 6S (clayey loam)) (*p* < 0.05) (Fig. 3).

## 4 Discussion

### 4.1 Pharmaceutical fate in soils

In agreement with previous research, carbamazepine was found to be persistent in all soil types (Monteiro and Boxall, 2009, Williams et al., 2006). Conversely a decline in radioactivity was measured in the diclofenac study, possible reasons for this include volatilisation and/or mineralisation (Fig. 1, Supplementary Figure 1). The K_d_ values fell within the ranges found in previous research for carbamazepine (0.49–37 L/kg (Drillia et al., 2005)) and diclofenac (1.21–17.72 L/kg, (Xu et al., 2009)) however for fluoxetine values were lower than previously reported (992–2546 L/kg, (Kwon and Armbrust, 2008)). Other than research primarily on veterinary antibiotics (Heise et al., 2006, Schmidt et al., 2008) this is some of the first published work to demonstrate that human pharmaceuticals can form irreversibly bound residues with soil and the degree of NER can be influenced by soil type (Supplementary Figure 1 and 2). Previous work has shown that non extractable pesticide residues remain available for uptake by earthworms (Gevao et al., 2001) and thus may be contributing to the uptake observed in this study.

The test soils selected for this study included soils with similar classifications, for example soil 2.1 and 2.3 are both ‘silty sand’ and soil 2.4 and 6S are both ‘clayey loam’. However, the behaviour of the pharmaceuticals in soils within the same class was quite different. For the chemicals that were unionised in the test system, carbamazepine and orlistat, porewater concentrations were greater in the silty sand soil that had the lowest organic content (soil 2.3 < soil 2.1). This would suggest hydrophobic interactions with the organic matter are driving the sorption process and thus the bioavailable fraction in the pore water. For the cationic pharmaceutical, fluoxetine, where it has been suggested that sorption to soil is regulated by the cation exchange sites present on the clay minerals and organic matter (Droge and Goss, 2013), higher pore water concentrations were measured in soils with lower CEC within the same soil type (soil 2.3 < soil 2.1 and soil 6S < soil 2.4). This would suggest that the fate of pharmaceuticals in soils is due to a combination of soil and pharmaceutical properties.

To the best of our knowledge, this is some of the first research to demonstrate that soil pH can change after addition of pharmaceuticals to the soil matrices. Both pharmaceutical physico-chemical properties and soil type appear to influence the degree of pH change, as changes in comparison to the controls and over time were not consistent across all treatment combinations (Fig. 2). Further analysis should explore this with a wider range of chemicals and soil types. The environment comprises of a wide range of ionisable chemicals and different soil types and these initial results may have considerable impact on environmental modelling scenarios, which currently do not account for changes in pH. Changes in soil pH may have significant effects on the fate of chemicals in the terrestrial environment by affecting processes such as sorption, leaching and degradation and should be considered in a modelling framework (Franco et al., 2009).

### 4.2 Relationships between soil and pore water properties with earthworm uptake

Regression analysis between various soil properties and BSAF values failed to highlight key factors which may be responsible for pharmaceutical uptake into earthworms. Previously clay and organic matter content have been shown to influence bioavailability of organic pollutants in soils (Chung and Alexander, 1998, Weber and Weed, 1968, White et al., 1997). Specifically, research has shown greater uptake into earthworms of the neutral organic compound phenanthrene in soils with higher clay content (White et al., 1997) however this was not observed with soil BSAF values calculated in this study.

This study used soils with an environmentally realistic pH range (6.6–8.2). Therefore, this may account for the lack of clear effect of soil pH on the uptake of pharmaceuticals into earthworms as the pH range was fairly small. Where notable differences in BSAFs were observed in the diclofenac study (Table 3), diclofenac was always extensively ionised (>99% Supplementary Table 3) and no relationship between BSAF and soil pH was found. Additional studies could explore pharmaceutical exposure in soils with a wider pH range as research has shown *E. fetida* can survive in soils as low as pH 4.3 (Sims and Gerard, 1999) which may elucidate relationships between pharmaceutical accumulation and pH effects such as those demonstrated in the aquatic environment (Nakamura et al., 2008).

As clear relationships between soil properties and earthworm BSAFs were unable to be found, it would suggest earthworm uptake is a complex interaction of a variety of factors and processes and does not exclusively rely on a single soil parameter. In addition, previous research has shown the ingestion of soil particles plays a very minor role in the accumulation of chemicals (log K_ow_ < 6) in earthworms (Jager et al., 2003, Vijver et al., 2003). Instead, for a large proportion of chemicals, uptake via diffusion across the earthworm skin dominates. Therefore, understanding how pore water properties relate to earthworm uptake and BCFs may be a more appropriate approach to evaluate differences in pharmaceutical accumulation between soil types. Highest internal concentrations were observed in exposures which had the highest pore water concentration of the respective pharmaceutical and therefore would suggest the bioavailability of chemicals in pore water is a limiting factor in earthworm uptake. For all pharmaceuticals, this was in the silt sand soil (soil 2.1), whilst the clayey loam soil (soil 2.4) generally had the lowest pore water concentrations (Fig. 1). However, high internal concentrations at the end of the exposure do not necessarily translate into highest BCFs. A BCF is a net result of competing rates of uptake and elimination and therefore should not be affected by the exposure medium concentration (Arnot and Gobas, 2006). Additional studies are required to elucidate whether earthworm uptake of pharmaceuticals is independent of exposure medium concentration and to explore relationships between BCF and pore water properties such as pH and dissolved organic matter.

Clearly many factors and processes in both the pore water and soil are governing the fate and subsequent uptake of pharmaceuticals into earthworms as current attempts to single out principal factors are yet to be successful. However, considering uptake as a combination of both soil and pore water parameters may offer a better explanation. Results showed increased chemical uptake by earthworms in soils which had decreasing soil organic matter (SOM). This could be explained by the presence of SOM decreasing the proportion of the chemical in pore water via sorption interactions which in turn reduces potential for uptake. Our results tend to agree that decreasing SOM leads to higher pore water concentrations of the pharmaceuticals (Supplementary Figure 3). For fluoxetine we saw a marked decrease in pore water concentration when there was an increase in organic carbon (OC) content of the test soils. Relationships presented in Supplementary Figure 3 also show an increase in OC corresponds to a decrease in BCFs for the various soils and thus fits with previous research findings that the SOM is regulating the bioavailable fraction of pharmaceuticals in the pore water. This is also shown in the diclofenac exposure, although to a lesser extent, with weak correlations especially between BCF and OC (Supplementary Figure 3).

For the neutral pharmaceuticals, orlistat and carbamazepine, an increase in organic carbon content still followed a decrease in pore water concentration. However, in contrast a decrease in pore water concentration corresponded to an increase in BCF (Supplementary Figure 3). There was a weak correlation between increasing BCF and increasing OC content of the soils such that it could be inferred that BCF is inversely related to pore water concentration for the neutral chemicals and inversely related to OC content for the acid and basic pharmaceuticals (Supplementary Figure 3). Clearly, complex interactions exist between SOM, pore water concentrations and BCFs. Further experiments, using a wider variety of soil types, would allow for appropriate exploration and conclusions to be drawn. Specifically, for pharmaceuticals present in their unionised form, where hydrophobic interactions dominate the sorption process, future studies need to take into account how the “hard” carbon fraction of the soil influences the bioavailability of pharmaceuticals. Hard carbon materials such as soot and black carbon have been previously demonstrated to influence organic chemical bioavailability in water-sediment systems (Cornelissen and Gustafsson, 2005, Rust et al., 2004, Tang et al., 2007).

As one single soil type did not generate the largest BCFs for all pharmaceuticals, our results demonstrate that earthworm uptake is both a factor of soil type (including soil and pore water parameters) as well as pharmaceutical physico-chemical properties. However, it is clear that for some pharmaceuticals the influence of soil type on the uptake and accumulation of pharmaceuticals is more significant (i.e. diclofenac) than for others (i.e. carbamazepine). Exposure in the terrestrial system is a dynamic process and the availability of chemicals to organisms is highly changeable. Whilst different soil types may affect the uptake and accumulation of some chemicals, BCF and BSAF results presented in this study suggest that the uptake of pharmaceuticals are less influenced by soil chemistry.

Information on how soil properties can affect chemical uptake is important in terms of both risk assessment and modelling. Currently used, generalised models are unlikely to accurately represent the potential uptake and risk associated with soil-borne contaminants and, as our research shows, numerous factors are involved in determining uptake. For modelling, a better understanding of biological factors influencing the uptake of chemicals residing in soils is important to accurately estimate the bioaccumulation potential. Additional work needs to explore the effect of changing pH in the earthworm tissue and soil samples on the uptake of ionisable chemicals and the subsequent implications of this for exposure modelling scenarios. Specifically, changes in earthworm tissue pH may result in wider implications involving pH dependent toxicity such as the ion trap phenomena previously observed in algal cells (Neuwoehner and Escher, 2011), or negative effects on earthworm internal environments. However, as it is not clear which factors specifically lead to pH change, further studies are needed to quantify and qualify these complicated processes.

This study represents the first attempt to evaluate the complex interplay between pharmaceutical chemical properties and soil chemical properties and how these govern potential exposure scenarios for a critical terrestrial organism. While there are many confounding complexities and unanswered questions this work represents a first important step in understand the terrestrial fate of pharmaceuticals, a critical component in understanding environmental risk.

Detailed information on extraction procedures together with extraction recoveries, a mass balance accounting for all radioactivity in the experiment and information on the ionisation state of the pharmaceuticals are provided in the Supplementary material.

## Figures and Tables

**Table 1:** Test pharmaceutical physico-chemical properties.

Pharmaceutical	Class	CAS^a^	Molecular weight (g mol^−1^)	Log K_ow_^b^	Acid/Base	pKa^c^	Specific activity (GBq mmol^−1^)
Carbamazepine	Anti-epileptic	298-46-4	236.30	2.25	Neutral	N/A	0.74
Diclofenac	Anti-inflammatory	15307-79-6	318.13	4.02	Acid	4.12	2.29
Fluoxetine	Anti-depressant	54910-89-3	345.80	4.65	Base	9.53	2.04
Orlistat	Weight loss aid	96829-58-2	497.74	8.19	Neutral	N/A	2.05

a CAS obtained from the Chemical Abstracts Service.

b Log Kow values obtained from KOWWIN v. 1.68 database, USEPA EPI suite 4.1 programme.

c pKa values were predicted using the University of Georgia SPARC database v. 4.2 (http://ibmlc2.chem.uga.edu/sparc) Accessed: 25/05/2012.

**Table 2:** Soil properties for the standard test LUFA Speyer soils. Mean values of different batch analyses are provided ± standard deviation (SD).

Standard soil type	2.1	2.3	2.4	5M	6S
Organic carbon in % C	0.7 ± 0.1	0.9 ± 0.1	2.3 ± 0.3	1.0 ± 0.2	1.6 ± 0.1
Nitrogen in % N	0.05 ± 0.01	0.08 ± 0.02	0.2 ± 0.04	0.1 ± 0.02	0.2 ± 0.02
pH value (0.01 M CaCl_2_)	5.1 ± 0.3	6.8 ± 0.2	7.2 ± 0.2	7.3 ± 0.1	7.1 ± 0.1
Cation exchange capacity (meq/100 g)	4.3 ± 0.5	10.9 ± 1.1	31.4 ± 4.6	16.6 ± 2.8	27.2 ± 1.4
Soil type	Silty sand	Silty sand	Clayey loam	Loamy sand	Clayey loam
Water holding capacity (g/100 g)	31.1 ± 2.1	37.3 ± 1.8	44.1 ± 1.2	39.5 ± 2.9	40.5 ± 2.1
Particle size (mm) distribution according to USDA (%)					
<0.002	2.8 ± 1.1	8.5 ± 1.7	25.9 ± 2.1	11.1 ± 1.2	40.5 ± 2.1
0.002–0.05	10.2 ± 1.8	28.4 ± 4.5	40.5 ± 1.0	29.7 ± 2.8	35.0 ± 2.9
0.05–2.0	87.0 ± 1.5	63.1 ± 5.0	33.6 ± 1.8	59.2 ± 3.2	24.5 ± 3.5

**Table 3:** Results from minimised design experiments in five soil types showing average measured concentrations in *E. fetida* (n = 6) at the end of 21 d uptake phase (*C*_*t1*_) and 21 d depuration phase (*C*_*t2*_) and average concentration (n = 3) of pharmaceutical in the pore water during the uptake phase (*C*_*pw*_) (±standard deviation). Calculated uptake (*k_1_*) and depuration rates (*k_2_*) are presented along with pore water based BCF values derived using the minimised design approach. Soil/water adsorption coefficients (K_d_) are also provided with soil BSAF estimates based on K_d_ values.

Soil type		*C*_*t1*_ Bq/g (internal)	*C*_*t2*_ Bq/g (internal)	*C*_*pw*_ (Bq/mL)	*k*_*2*_ (dep. Rate) (d^−1^)		*k*_*1*_ (uptake rate) (mL/g d^−1^)		Pore water BCF	Soil K_d_ (average 21 d)	Soil BSAF
**Carbamazepine**											
2.1	76.13 ± 13.33		1.49 ± 0.68	59.78 ± 14.61		0.187	0.24	1.30		1.34	0.97
2.3	27.94 ± 6.11		0.17 ± 0.03	18.37 ± 3.57		0.243	0.37	1.53		3.87	0.40
2.4	25.79 ± 2.94		0.28 ± 0.74	16.24 ± 2.27		0.215	0.35	1.61		4.45	0.36
5M	34.49 ± 3.09		0.52 ± 0.52	33.37 ± 5.58		0.200	0.21	1.05		2.20	0.48
6S	35.10 ± 4.82		1.22 ± 0.36	23.35 ± 6.03		0.160	0.25	1.56		3.44	0.45
**Diclofenac**											
2.1	413.80 ± 166.08		233.65 ± 141.58	33.28 ± 13.72		0.027	0.77	28.56		6.88	4.15
2.3	34.12 ± 13.76		31.67 ± 12.88	31.50 ± 11.67		0.004	0.05	15.04		7.25	2.07
2.4	31.61 ± 9.61		26.64 ± 8.74	10.85 ± 1.92		0.008	0.15	18.53		18.37	1.01
5M	34.59 ± 3.52		34.14 ± 11.51	38.20 ± 14.80		0.001	0.04	69.57		5.63	12.36
6S	67.99 ± 11.95		42.29 ± 22.54	25.64 ± 9.93		0.023	0.16	7.02		6.37	1.10
**Fluoxetine**											
2.1	61.94 ± 8.65		5.52 ± 0.96	3.33 ± 0.58		0.115	2.35	20.42		55.48	0.37
2.3	45.27 ± 5.81		7.82 ± 2.36	2.77 ± 0.42		0.084	1.65	19.74		64.85	0.32
2.4	22.46 ± 3.55		5.41 ± 1.78	2.10 ± 0.29		0.068	0.96	14.09		71.44	0.20
5M	34.70 ± 6.66		10.09 ± 3.69	2.55 ± 0.29		0.059	1.13	19.18		64.06	0.30
6S	28.91 ± 12.23		2.97 ± 0.81	1.91 ± 0.61		0.108	1.83	16.89		58.17	0.29
**Orlistat**											
2.1	116.83 ± 19.03		57.15 ± 6.97	7.50 ± 3.22		0.034	1.04	30.50		28.99	1.05
2.3	56.91 ± 8.12		47.04 ± 9.52	2.83 ± 1.45		0.009	1.05	115.88		75.10	1.54
2.4	35.43 ± 6.14		26.03 ± 5.86	1.78 ± 0.78		0.015	1.10	74.95		110.01	0.75
5M	56.88 ± 15.30		26.61 ± 13.73	2.60 ± 1.58		0.036	1.49	41.13		84.59	0.49
6S	37.93 ± 7.12		32.68 ± 14.15	3.98 ± 1.37		0.007	0.49	68.86		51.30	1.34

## References

[bib1] Arnold K.E., Brown A.R., Ankley G.T., Sumpter J.P. (2014). Medicating the environment: assessing risks of pharmaceuticals to wildlife and ecosystems. Philos. Trans. R. Soc. B Biol. Sci..

[bib2] Arnot J.A., Gobas F.A.P.C. (2006). A review of bioconcentration factor (BCF) and bioaccumulation factor (BAF) assessments for organic chemicals in aquatic organisms. Environ. Rev..

[bib3] Berge A., Vulliet E. (2015). Development of a method for the analysis of hormones and pharmaceuticals in earthworms by quick, easy, cheap, effective, rugged and safe (QuEChERS) extraction followed by liquid chromatography-tandem mass spectrometry (LC-MS/MS). Anal. Bioanal. Chem..

[bib4] Carter L.J., Ashauer R., Ryan J.J., Boxall A.B.A. (2014). Minimised bioconcentration tests: a useful tool for assessing chemical uptake into terrestrial and aquatic invertebrates?. Environ. Sci. Technol..

[bib5] Carter L.J., Garman C.D., Ryan J., Dowle A., Bergstrom E., Thomas-Oates J., Boxall A.B.A. (2014). Fate and uptake of pharmaceuticals in soil-earthworm systems. Environ. Sci. Technol..

[bib6] Chung N.H., Alexander M. (1998). Differences in sequestration and bioavailability of organic compounds aged in dissimilar soils. Environ. Sci. Technol..

[bib7] Cornelissen G., Gustafsson O. (2005). Prediction of large variation in biota to sediment accumulation factors due to concentration-dependent black carbon adsorption of planar hydrophobic organic compounds. Environ. Toxicol. Chem..

[bib8] Dalkmann P., Broszat M., Siebe C., Willaschek E., Sakinc T., Huebner J., Amelung W., Grohmann E., Siemens J. (2012). Accumulation of pharmaceuticals, enterococcus, and resistance genes in soils irrigated with wastewater for zero to 100 years in Central Mexico. PLoS One.

[bib9] Drillia P., Stamatelatou K., Lyberatos G. (2005). Fate and mobility of pharmaceuticals in solid matrices. Chemosphere.

[bib10] Droge S.T.J., Goss K.-U. (2013). Development and evaluation of a new sorption model for organic cations in soil: contributions from organic matter and clay minerals. Environ. Sci. Technol..

[bib11] Duran-Alvarez J.C., Becerril-Bravo E., Castro V.S., Jimenez B., Gibson R. (2009). The analysis of a group of acidic pharmaceuticals, carbamazepine, and potential endocrine disrupting compounds in wastewater irrigated soils by gas chromatography-mass spectrometry. Talanta.

[bib12] Edwards C.A. (2004). Earthworm Ecology.

[bib13] Fatta-Kassinos D., Vasquez M.I., Kuemmerer K. (2011). Transformation products of pharmaceuticals in surface waters and wastewater formed during photolysis and advanced oxidation processes – degradation, elucidation of byproducts and assessment of their biological potency. Chemosphere.

[bib14] Folch J., Lees M., Stanley G.H.S. (1957). A simple method for the isolation and purification of total lipides from animal tissues. J. Biol. Chem..

[bib15] Franco A., Fu W.J., Trapp S. (2009). Influence of soil pH on the sorption of ionizable chemicals: modeling advances. Environ. Toxicol. Chem..

[bib16] Gevao B., Mordaunt C., Semple K.T., Piearce T.G., Jones K.C. (2001). Bioavailability of nonextractable (bound) pesticide residues to earthworms. Environ. Sci. Technol..

[bib17] Heise J., Hoeltge S., Schrader S., Kreuzig R. (2006). Chemical and biological characterization of non-extractable sulfonamide residues in soil. Chemosphere.

[bib18] Jager T., Fleuren R., Hogendoorn E.A., De Korte G. (2003). Elucidating the routes of exposure for organic chemicals in the earthworm, Eisenia andrei (Oligochaeta). Environ. Sci. Technol..

[bib19] Jelic A., Gros M., Ginebreda A., Cespedes-Sanchez R., Ventura F., Petrovic M., Barcelo D. (2011). Occurrence, partition and removal of pharmaceuticals in sewage water and sludge during wastewater treatment. Water Res..

[bib20] Kinney C.A., Furlong E.T., Kolpin D.W., Burkhardt M.R., Zaugg S.D., Werner S.L., Bossio J.P., Benotti M.J. (2008). Bioaccumulation of pharmaceuticals and other anthropogenic waste indicators in earthworms from agricultural soil amended with biosolid or swine manure. Environ. Sci. Technol..

[bib21] Kinney C.A., Furlong E.T., Werner S.L., Cahill J.D. (2006). Presence and distribution of wastewater-derived pharmaceuticals in soil irrigated with reclaimed water. Environ. Toxicol. Chem..

[bib22] Kinney C.A., Furlong E.T., Zaugg S.D., Burkhardt M.R., Werner S.L., Cahill J.D., Jorgensen G.R. (2006). Survey of organic wastewater contaminants in biosolids destined for land application. Environ. Sci. Technol..

[bib23] Kodesova R., Grabic R., Kocarek M., Klement A., Golovko O., Fer M., Nikodem A., Jaksik O. (2015). Pharmaceuticals' sorptions relative to properties of thirteen different soils. Sci. Total Environ..

[bib24] Kwon J.-W., Armbrust K.L. (2008). Aqueous solubility, n-octanol-water partition coefficient, and sorption of five selective serotonin reuptake inhibitors to sediments and soils. Bull. Environ. Contam. Toxicol..

[bib25] Monteiro S.C., Boxall A.B.A. (2009). Factors affecting the degradation of pharmaceuticals in agricultural soils. Environ. Toxicol. Chem..

[bib26] Nakamura Y., Yamamoto H., Sekizawa J., Kondo T., Hirai N., Tatarazako N. (2008). The effects of pH on fluoxetine in Japanese medaka (Oryzias latipes): acute toxicity in fish larvae and bioaccumulation in juvenile fish. Chemosphere.

[bib27] Neuwoehner J., Escher B.I. (2011). The pH-dependent toxicity of basic pharmaceuticals in the green algae Scenedesmus vacuolatus can be explained with a toxicokinetic ion-trapping model. Aquat. Toxicol..

[bib28] Rust A.J., Burgess R.M., McElroy A.E., Cantwell M.G., Brownawell B.J. (2004). Influence of soot carbon on the bioaccumulation of sediment-bound polycyclic aromatic hydrocarbons by marine benthic invertebrates: an interspecies comparison. Environ. Toxicol. Chem..

[bib29] Schmidt B., Ebert J., Lamshoeft M., Thiede B., Schumacher-Buffel R., Ji R., Corvini P.F.X., Schaeffer A. (2008). Fate in soil of C-14-sulfadiazine residues contained in the manure of young pigs treated with a veterinary antibiotic. J. Environ. Sci. Health Part B Pestic. Food Contam. Agric. Wastes.

[bib30] Shore R.F., Taggart M.A., Smits J., Mateo R., Richards N.L., Fryday S. (2014). Detection and drivers of exposure and effects of pharmaceuticals in higher vertebrates. Philos. Trans. R. Soc. B Biol. Sci..

[bib31] Siemens J., Huschek G., Siebe C., Kaupenjohann M. (2008). Concentrations and mobility of human pharmaceuticals in the world's largest wastewater irrigation system, Mexico City-Mezquital Valley. Water Res..

[bib32] Sims R.W., Gerard B.M. (1999). Earthworms: Notes for the Identification of British Species, Synopses of the British Fauna.

[bib33] Tang J., Petersen E.J., Huang Q., Weber W.J. (2007). Development of engineered natural organic sorbents for environmental applications: 3. Reducing PAH mobility and bioavailability in contaminated soil and sediment systems. Environ. Sci. Technol..

[bib34] ter Laak T.L., Gebbink W.A., Tolls J. (2006). Estimation of soil sorption coefficients of veterinary pharmaceuticals from soil properties. Environ. Toxicol. Chem..

[bib35] Vijver M.G., Vink J.P.M., Miermans C.J.H., van Gestel C.A.M. (2003). Oral sealing using glue: a new method to distinguish between intestinal and dermal uptake of metals in earthworms. Soil Biol. Biochem..

[bib36] Weber J.B., Weed S.B. (1968). Adsorption and desorption of diquat paraquat and prometone by montmorillonitic and kaolinitic clay minerals. Soil Sci. Soc. Am. Proc..

[bib37] White J.C., Kelsey J.W., Hatzinger P.B., Alexander M. (1997). Factors affecting sequestration and bioavailability of phenanthrene in soils. Environ. Toxicol. Chem..

[bib38] Williams C.F., Williams C.E., Adamsen E.J. (2006). Sorption-desorption of carbamazepine from irrigated soils. J. Environ. Qual..

[bib39] Xu J., Wu L.S., Chang A.C. (2009). Degradation and adsorption of selected pharmaceuticals and personal care products (PPCPs) in agricultural soils. Chemosphere.

