# Supplementary material for: Effects of soil properties on the uptake of pharmaceuticals into earthworms
Source: Environ Pollut. 2016 Jun;213:922–31. doi: 10.1016/j.envpol.2016.03.044 (PMC4894142; doi:10.1016/j.envpol.2016.03.044)
Supplement: Supplementary file 1 [file mmc1.docx]

# Supplementary material

# Effects of soil properties on the uptake of pharmaceuticals into earthworms

Laura J. Carter^1^, Jim J. Ryan^2^, Alistair B.A. Boxall^1^

^1^ – Environment Department, University of York, Heslington, York, UK, YO10 5DD

^2^ – EHS Technical CoE, GlaxoSmithKline, Ware, UK, SG12 0DP

Number of tables: 3

Number of figures: 3

Additional text: Extraction of pharmaceuticals from soil, earthworm and pore water

Supplementary Table 1 *Eisenia fetida* mass at beginning of experiment

Average earthworm mass (n=6) for each of the treatments included in the experiment (± standard deviation). The overall *E. fetida* mass was 299 mg with a standard deviation of 54 mg. ANOVA revealed no significant difference in the earthworm mass between treatments (carbamazepine, diclofenac, fluoxetine and orlistat) p = 0.111)

|  | Carbamazepine | Diclofenac | Fluoxetine | Orlistat |
| --- | --- | --- | --- | --- |
| Soil 2.1 | 0.320 ± 0.03 | 0.311 ± 0.06 | 0.285 ± 0.06 | 0.333 ± 0.06 |
| Soil 2.3 | 0.307 ± 0.03 | 0.318 ± 0.07 | 0.316 ± 0.06 | 0.302 ± 0.06 |
| Soil 2.4 | 0.266 ± 0.03 | 0.272 ± 0.06 | 0.339 ± 0.07 | 0.318 ± 0.06 |
| Soil 5M | 0.270 ± 0.02 | 0.296 ± 0.07 | 0.295 ± 0.04 | 0.300 ± 0.06 |
| Soil 6S | 0.269 ± 0.03 | 0.279 ± 0.05 | 0.294 ± 0.04 | 0.289 ± 0.04 |
|  |  |  |  |  |
| **Average** | **0.286 ± 0.03** | **0.295 ± 0.02** | **0.306 ± 0.02** | **0.309 ± 0.02** |

Supplementary Table 2 Extraction recoveries

Experiments were carried out to determine the recovery of carbamazepine, diclofenac, fluoxetine and orlistat from five soil types using solvent extraction. For each soil type and each pharmaceutical 5 g of soil was prepared in triplicate and spiked with a known amount of pharmaceutical compound. Average recoveries for each soil type and compound provided ± standard deviation.

| Pharmaceutical | Soil type | Solvent | 1 x extraction (% recovery ± S.D.) | 2 x extraction (% recovery ± S. D.) |
| --- | --- | --- | --- | --- |
| Carbamazepine | 2.1 | Methanol | 85.68 ± 10.12 | 93.65 ± 12.22 |
| Carbamazepine | 2.3 | Methanol | 79.25 ± 6.71 | 86.79 ± 5.00 |
| Carbamazepine | 2.4 | Methanol | 84.88 ± 1.94 | 92.61 ± 2.93 |
| Carbamazepine | 5M | Methanol | 86.50 ± 2.84 | 93.00 ± 2.99 |
| Carbamazepine | 6S | Methanol | 83.15 ± 2.81 | 94.72 ± 3.96 |
| Diclofenac | 2.1 | Ethyl Acetate | 93.05 ± 2.31 | 93.52 ± 2.00 |
| Diclofenac | 2.3 | Ethyl Acetate | 90.71 ± 1.79 | 94.67 ± 1.97 |
| Diclofenac | 2.4 | Ethyl Acetate | 90.11 ± 1.71 | 92.36 ± 0.70 |
| Diclofenac | 5M | Ethyl Acetate | 77.04 ± 1.55 | 86.37 ± 4.31 |
| Diclofenac | 6S | Ethyl Acetate | 83.72 ± 5.52 | 90.17 ± 5.89 |
| Fluoxetine | 2.1 | Acetonitrile:Water (7:3) | 73.96 ± 5.19 | 82.19 ± 5.07 |
| Fluoxetine | 2.3 | Acetonitrile:Water (7:3) | 66.16 ± 2.21 | 76.52 ± 1.48 |
| Fluoxetine | 2.4 | Acetonitrile:Water (7:3) | 61.33 ± 0.60 | 74.93 ± 1.31 |
| Fluoxetine | 5M | Acetonitrile:Water (7:3) | 66.26 ± 0.80 | 78.06 ± 1.65 |
| Fluoxetine | 6S | Acetonitrile:Water (7:3) | 36.78 ± 0.68 | 72.43 ± 1.61 |
| Orlistat | 2.1 | Acetonitrile | 84.36 ± 4.52 | 88.11 ± 2.19 |
| Orlistat | 2.3 | Acetonitrile | 82.00 ± 2.37 | 82.84 ± 1.94 |
| Orlistat | 2.4 | Acetonitrile | 79.07 ± 1.75 | 82.25 ± 2.04 |
| Orlistat | 5M | Acetonitrile | 80.57 ± 2.59 | 82.28 ± 1.00 |
| Orlistat | 6S | Acetonitrile | 81.83 ± 3.69 | 83.13 ± 3.62 |

Supplementary Table 3 Extent of diclofenac and fluoxetine ionisation in earthworm and soil

Percentage of ionised and neutral species in different soil types and worm samples for diclofenac and fluoxetine exposure at 0H (start pH) and 21 d (end pH) are provided below.

| **Diclofenac** | |  |  |  |  |  |  |  |  |
| --- | --- | --- | --- | --- | --- | --- | --- | --- | --- |
|  | soil | | | |  | soil | | | |
|  | **start pH** | **anion:neutral** | **% neutral** | **% ionic** |  | **end pH** | **anion:neutral** | **% neutral** | **% ionic** |
| Soil 2.1 | 6.70 | 377.28 | 0.2644 | 99.74 |  | 6.69 | 368.69 | 0.2705 | 99.73 |
| Soil 2.3 | 7.03 | 812.83 | 0.1229 | 99.88 |  | 7.00 | 758.58 | 0.1317 | 99.87 |
| Soil 2.4 | 7.72 | 4011.75 | 0.0249 | 99.98 |  | 7.52 | 2531.24 | 0.0395 | 99.96 |
| Soil 5M | 8.14 | 10471.29 | 0.0095 | 99.99 |  | 8.12 | 10000.00 | 0.0100 | 99.99 |
| Soil 6S | 7.92 | 6358.19 | 0.0157 | 99.98 |  | 7.90 | 5979.52 | 0.0167 | 99.98 |
|  |  |  |  |  |  |  |  |  |  |
|  | worm | | | |  | worm | | | |
|  | **end uptake pH** | **anion:neutral** | **% neutral** | **% ionic** |  | **end dep pH** | **anion:neutral** | **% neutral** | **% ionic** |
| Soil 2.1 | 6.84 | 518.80 | 0.1924 | 99.81 |  | 6.86 | 551.65 | 0.1809 | 99.82 |
| Soil 2.3 | 6.68 | 363.08 | 0.2747 | 99.73 |  | 6.78 | 459.20 | 0.2173 | 99.78 |
| Soil 2.4 | 6.91 | 614.23 | 0.1625 | 99.84 |  | 6.99 | 744.16 | 0.1342 | 99.87 |
| Soil 5M | 6.89 | 582.10 | 0.1715 | 99.83 |  | 6.83 | 514.83 | 0.1939 | 99.81 |
| Soil 6S | 6.79 | 465.59 | 0.2143 | 99.79 |  | 6.96 | 683.91 | 0.1460 | 99.85 |

| **Fluoxetine** | |  |  |  |  |  |  |  |  |
| --- | --- | --- | --- | --- | --- | --- | --- | --- | --- |
|  | soil | | | |  | soil | | | |
|  | **start pH** | **anion:neutral** | **% neutral** | **% ionic** |  | **end pH** | **anion:neutral** | **% neutral** | **% ionic** |
| Soil 2.1 | 6.19 | 2187.76 | 0.05 | 99.95 |  | 6.84 | 493.55 | 0.20 | 99.80 |
| Soil 2.3 | 6.88 | 450.13 | 0.22 | 99.78 |  | 7.16 | 236.23 | 0.42 | 99.58 |
| Soil 2.4 | 7.21 | 207.33 | 0.48 | 99.52 |  | 7.68 | 70.79 | 1.39 | 98.61 |
| Soil 5M | 8.10 | 26.92 | 3.58 | 96.42 |  | 8.35 | 15.14 | 6.20 | 93.80 |
| Soil 6S | 8.09 | 27.75 | 3.48 | 96.52 |  | 8.04 | 31.14 | 3.11 | 96.89 |
|  |  |  |  |  |  |  |  |  |  |
|  | worm | | | |  | worm | | | |
|  | **end uptake pH** | **anion:neutral** | **% neutral** | **% ionic** |  | **end dep pH** | **anion:neutral** | **% neutral** | **% ionic** |
| Soil 2.1 | 7.03 | 316.23 | 0.32 | 99.68 |  | 6.91 | 418.47 | 0.24 | 99.76 |
| Soil 2.3 | 6.83 | 503.11 | 0.20 | 99.80 |  | 6.66 | 503.11 | 0.20 | 99.80 |
| Soil 2.4 | 6.96 | 368.69 | 0.27 | 99.73 |  | 6.73 | 368.69 | 0.27 | 99.73 |
| Soil 5M | 6.81 | 530.88 | 0.19 | 99.81 |  | 6.66 | 530.88 | 0.19 | 99.81 |
| Soil 6S | 6.84 | 489.78 | 0.20 | 99.80 |  | 6.77 | 489.78 | 0.20 | 99.80 |

Supplementary Figure 1 Mass balance for treatments where non-extractable residues (NER) were detected (diclofenac)

Mass balance for diclofenac in fate study over 0 and 6 h, 1, 3, 7, 10, 14 and 21 d in 5 soil types. The amount of radioactivity measured in the solvent extraction (extractable) and combustion analysis (NER) for each soil type was calculated as a percentage of the measured radioactivity in the test system at the start of the fate study (0 h) with any remaining difference in radioactivity being unaccounted for. It is likely that the unaccounted for fraction would indicate mineralisation or volatilisation. Figures show percentage recovered from non-extractable residues (grey), solvent extractable residues (white) and unaccounted for (black) in comparison to radioactivity measured in the soil at 0H.


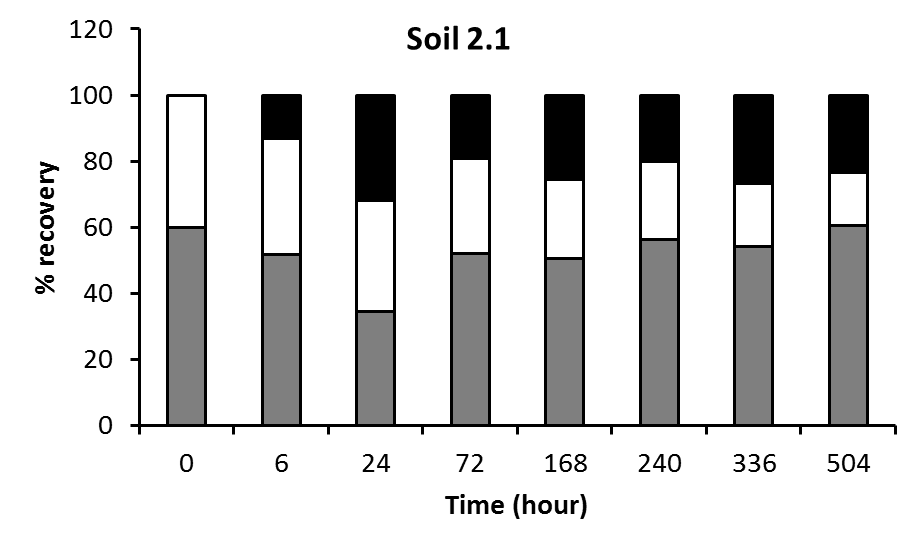

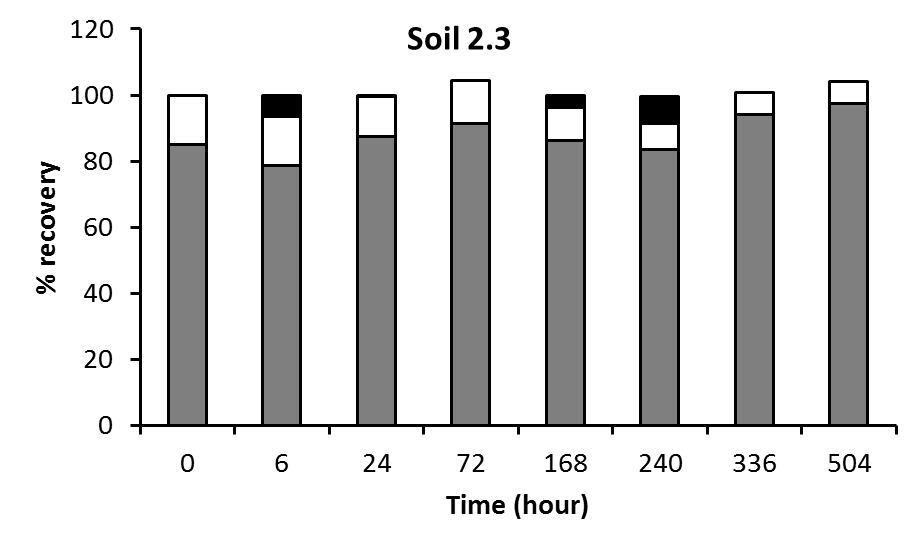

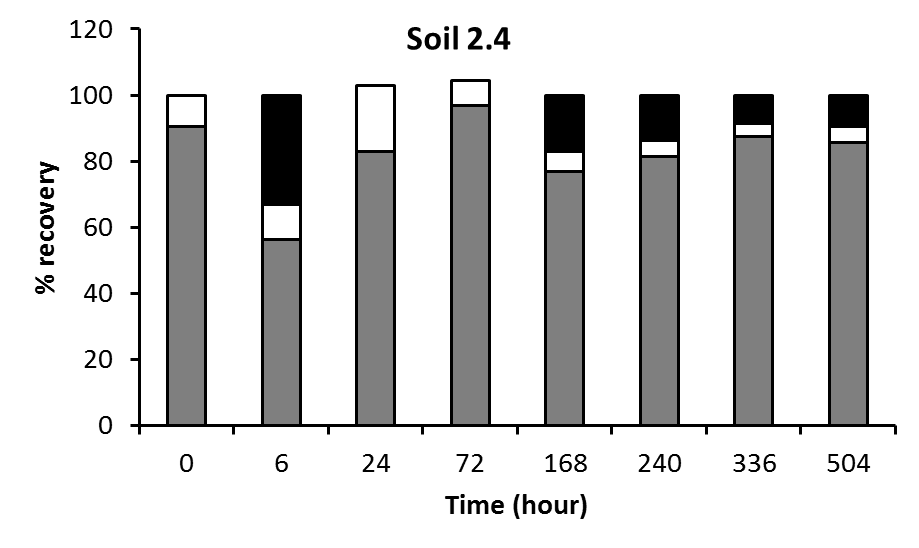

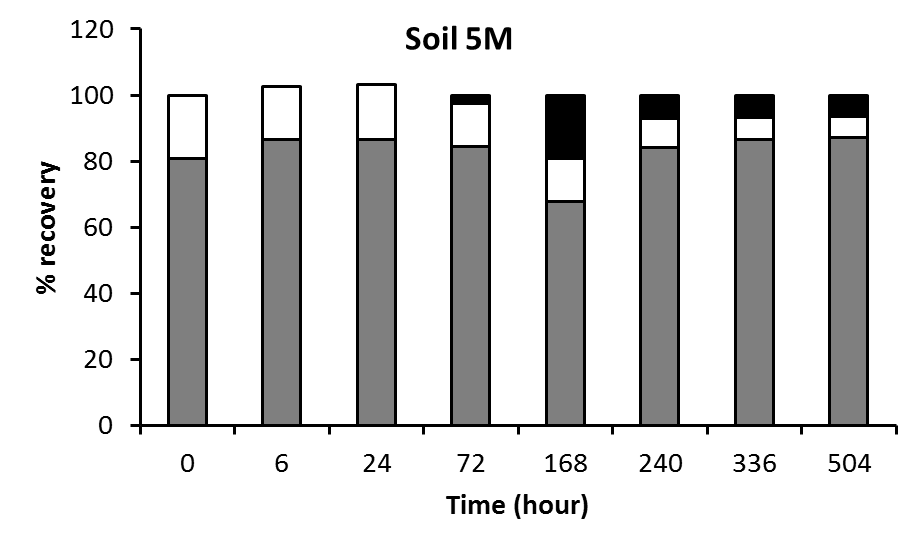

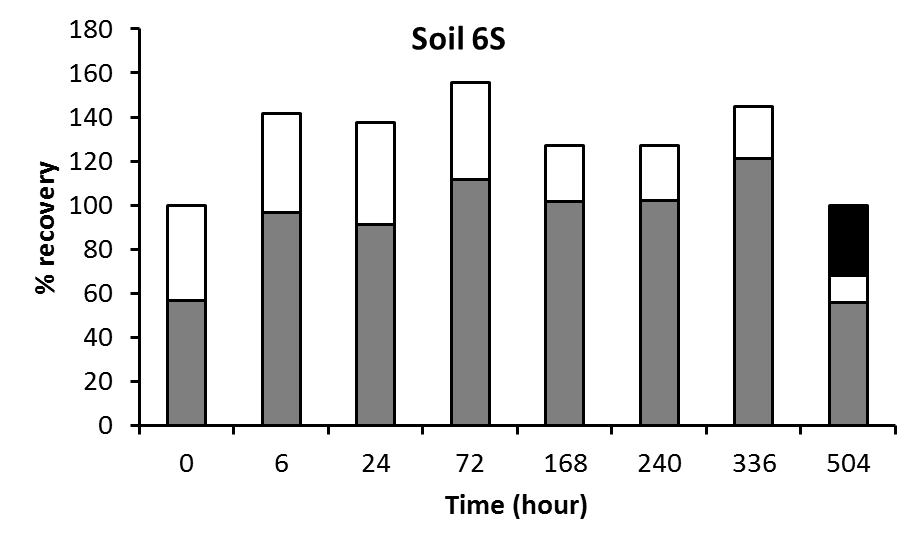


Supplementary Figure 2 Mass balance for treatments where non-extractable (NER) residues were detected (orlistat)

Mass balance for orlistat in fate study over 0 and 6 h, 1, 3, 7, 10, 14 and 21 d in 5 soil types. The amount of radioactivity measured in the solvent extraction (extractable) and combustion analysis (NER) for each soil type was calculated as a percentage of the measured radioactivity in the test system at the start of the fate study (0 h) with any remaining difference in radioactivity being unaccounted for. It is likely that the unaccounted for fraction would indicate mineralisation or volatilisation. Figures show percentage recovered from non-extractable residues (grey), solvent extractable residues (white) and unaccounted for (black) in comparison to radioactivity measured in the soil at 0H.


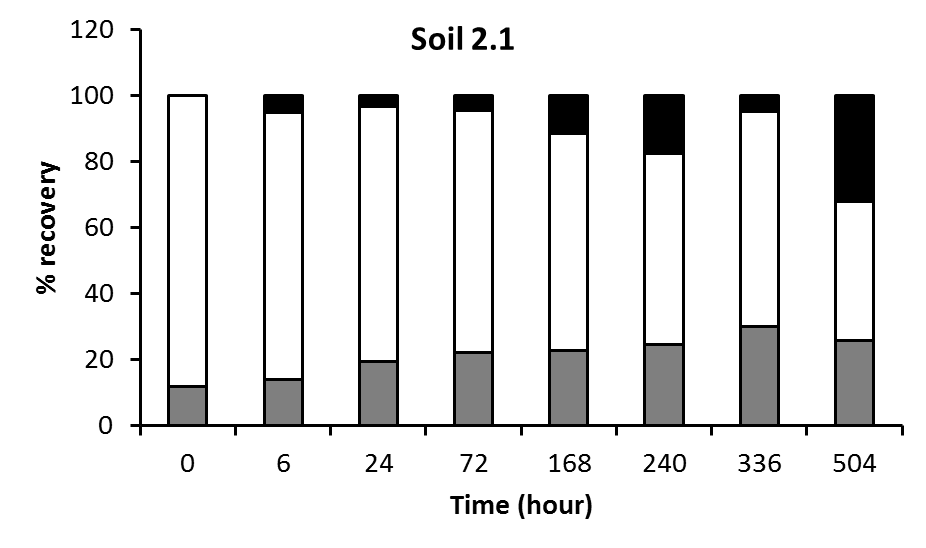

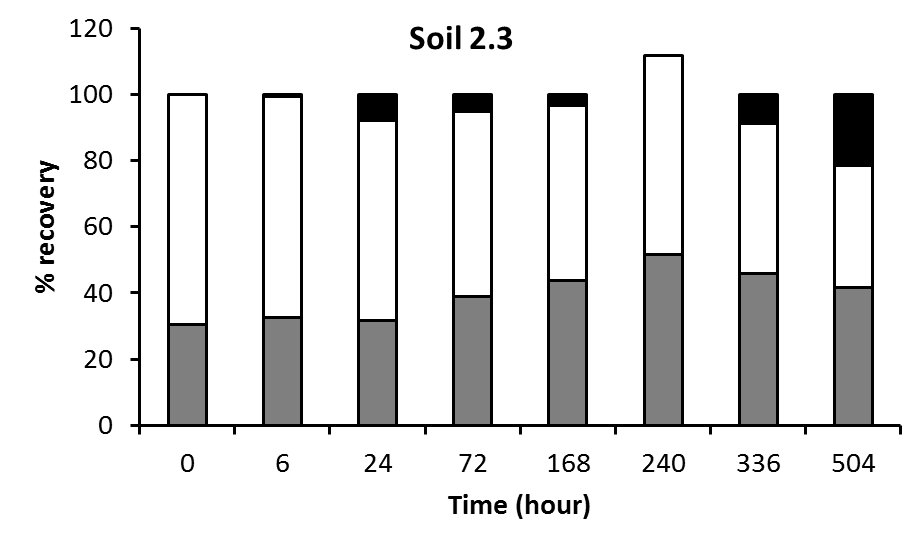

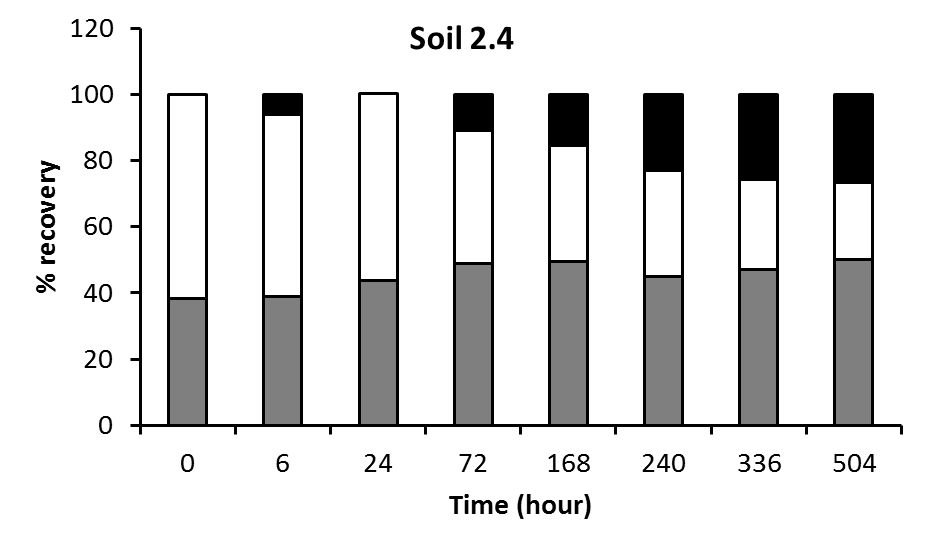

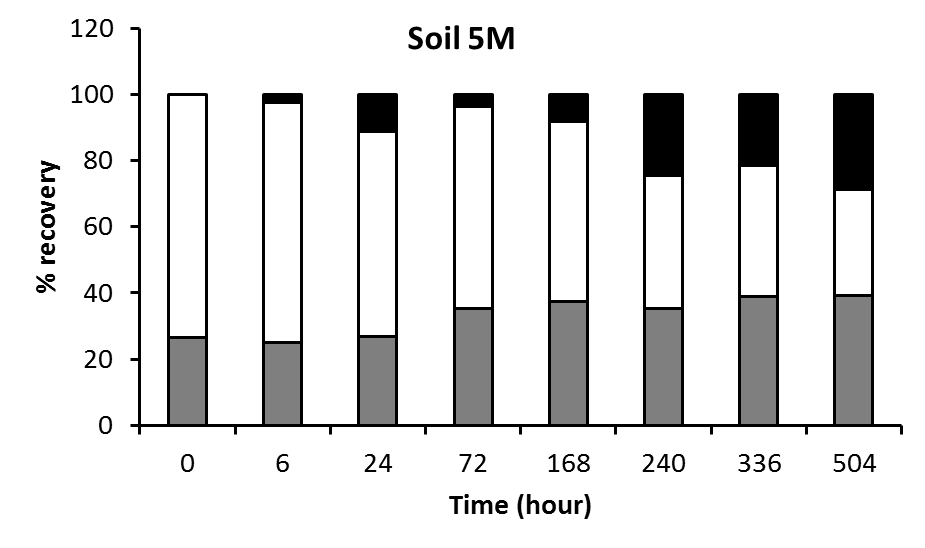

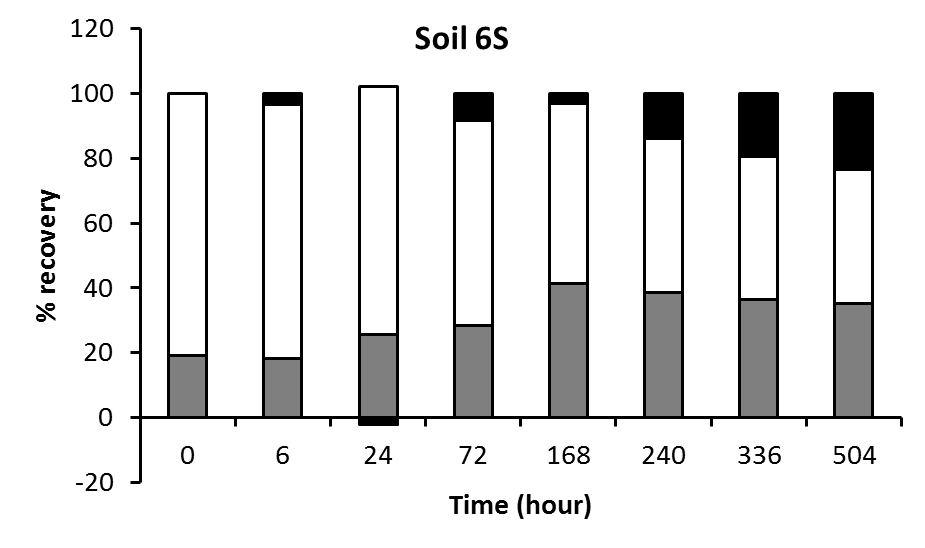


B

B

Supplementary Figure 3 Relationship between BCF, soil organic carbon and pore water concentrations

Correlations between organic carbon content, pore water concentration and bioconcentration factor for carbamazepine (CBZ), diclofenac (DCF), fluoxetine (FLX) and orlistat (ORL)


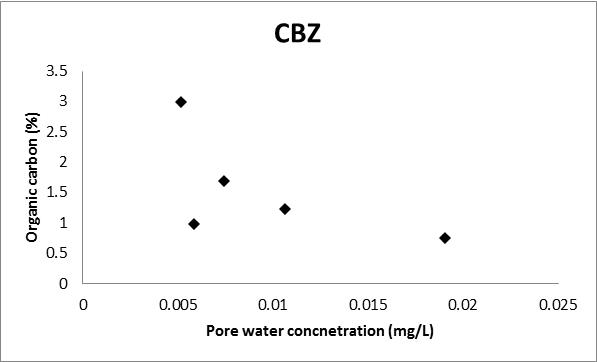

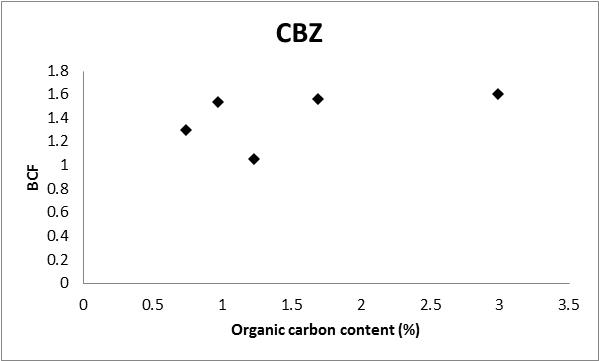

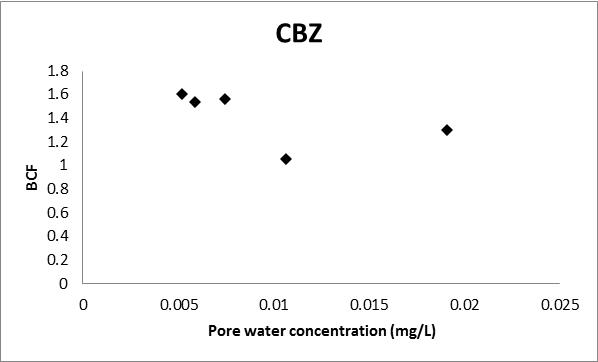


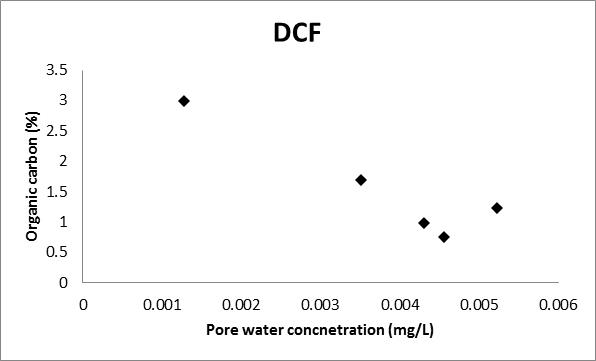

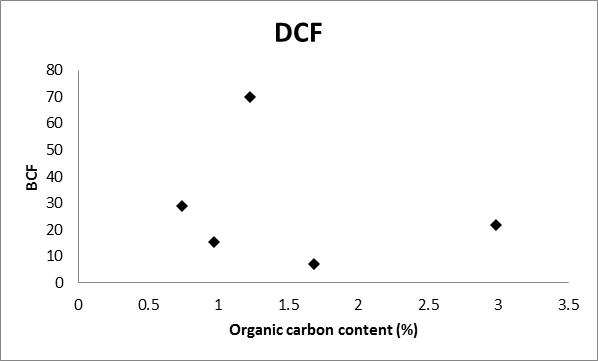

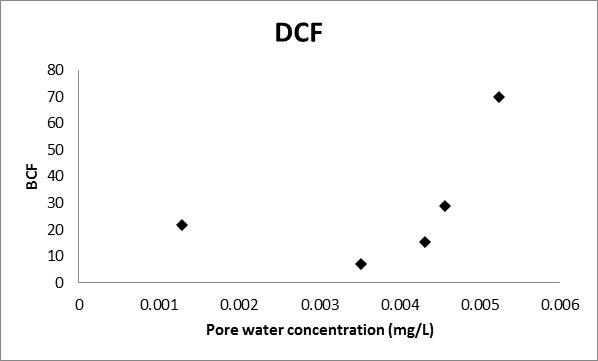


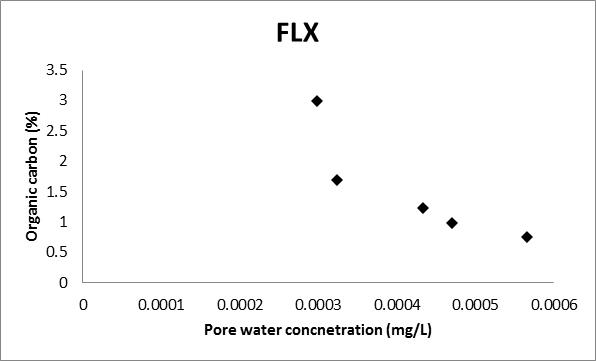

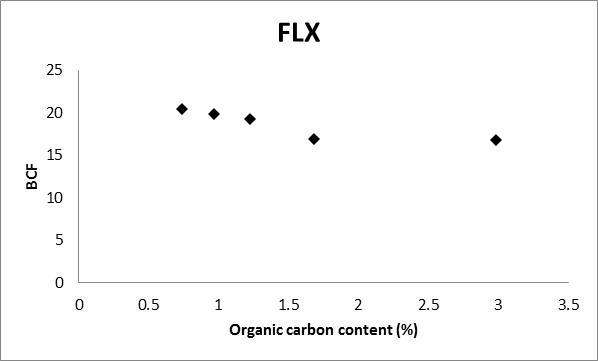

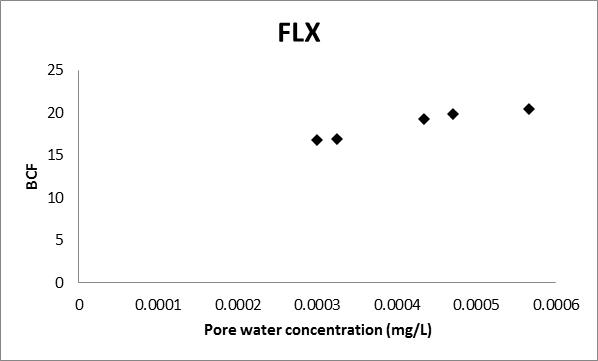


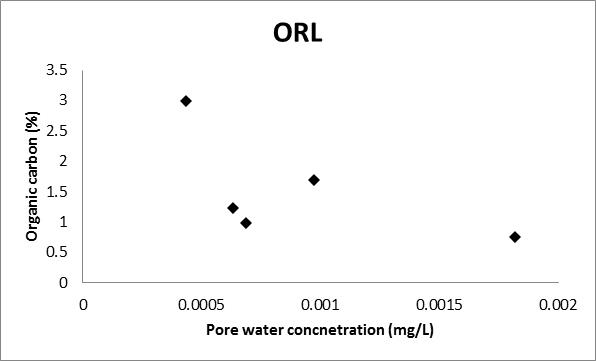

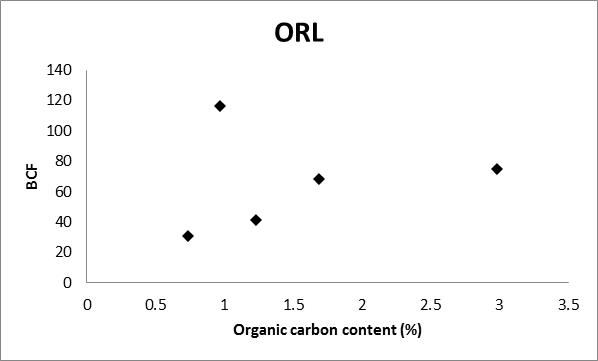

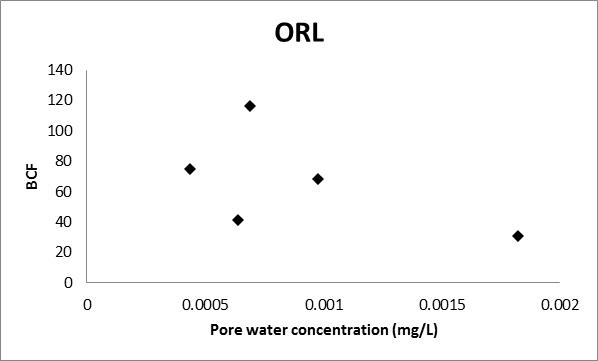


Preparation of samples for analysis

Soil pore water extraction:

To extract pore water, soil (25 ± 2g) was placed in a disposable syringe with a layer of 3cm of glass wool inserted into the bottom. The syringe was centrifuged for 40 minutes (2 x 20 minute runs) at 3000 RPM after which the pore water was collected from the bottom of the tube and transferred to a 2 mL microfuge tube. The microfuge tubes containing the sampled pore water were then further centrifuged at 12000 RCF for 4 minutes to sediment loose particles. A 500 µL sample of pore water was then added to 10 mL of EcoScint A scintillation cocktail for analysis.

Soil extraction:

For the carbamazepine study, 5 ± 0.5 g of soil was extracted twice for 45 minutes on a side to side shaker (250 oscillations min^-1^) with 2 x 10 mL of methanol. A similar method was used in the fluoxetine and orlistat studies except that a mixture of acetonitrile and water (7:3 v/v) and acetonitrile only were used as solvents, respectively. For the diclofenac study, 5 g samples of soil were extracted three times for 45 minutes with 3 x 10 mL ethyl acetate. Samples (1 mL) of extracts were then added to 10 mL of EcoScint A for analysis of the radioactivity present.

Soil combustion analysis:

Combustion analysis was performed using a Perkin Elmer 307 Sample Oxidiser. After solvent extraction, the dried soils were homogenised into a fine powder. Each soil sample was prepared in triplicate in combusto-cones where 300 ± 25 mg of soil was mixed with cellulose. After combustion consisting of a 1.5 minute burn per sample, the ^14^C carbon dioxide was trapped by a vapour phase reaction with CarboSorb E forming carbamate which was mixed with PermaFluor E + a scintillation cocktail ready for counting the radioactivity present on the Liquid Scintillation Counter (LSC). Regular checks were performed throughout the analysis to ensure the recovery of the samples remained above 97 %.

Earthworm extraction:

*E. fetida* were extracted by liquid extraction using the same solvents as for the soil extractions. For each worm 5 mL of solvent was added and the worm/solvent mix was homogenised for 5 minutes using a LabGen Series 7 homogeniser. The suspension was transferred to a glass test tube and the beaker was then rinsed with an additional 3 ml of solvent which was combined with the original suspension to give a total volume of 8 mL. This was centrifuged at 415 g for 30 minutes (CHRIST Rotational Vacuum-Concentrator RVC 2-33 CD) and a 1 mL sample of the supernatant was then added to 10 mL of EcoScint A for analysis of the radioactivity present.
